# Influence of Contaminants Mercury and PAHs on Somatic Indexes of the European Hake (*Merluccius merluccius*, L. 1758)

**DOI:** 10.3390/ani14202938

**Published:** 2024-10-11

**Authors:** Monica Panfili, Stefano Guicciardi o Guizzardi, Emanuela Frapiccini, Cristina Truzzi, Federico Girolametti, Mauro Marini, Alberto Santojanni, Anna Annibaldi, Silvia Illuminati, Sabrina Colella

**Affiliations:** 1 Institute for Marine Biological Resources and Biotechnologies, National Research Council (IRBIM-CNR), 60125 Ancona, Italy; monica.panfili@cnr.it (M.P.); stefano.guicciardioguizzardi@cnr.it (S.G.o.G.); mauro.marini@cnr.it (M.M.); alberto.santojanni@cnr.it (A.S.); sabrina.colella@cnr.it (S.C.); 2 Department of Life and Environmental Sciences, Università Politecnica delle Marche, 60131 Ancona, Italy; f.girolametti@staff.univpm.it (F.G.); a.annibaldi@staff.univpm.it (A.A.); s.illuminati@staff.univpm.it (S.I.)

**Keywords:** total mercury, PAHs, *Merluccius merluccius*, lipid content, seasonal variability, condition indexes, Adriatic Sea

## Abstract

**Simple Summary:**

Recent awareness highlights the significant impact of contaminants on the Mediterranean marine ecosystem and fishery resources. Monitoring these pollutants is crucial due to their accumulation in marine organisms and the health risks they pose through consumption. This study examines the levels of total mercury and PAHs in the muscle tissue of European hake from an important fishing ground in the Adriatic Sea. Seasonal and gender patterns as well as correlations with somatic indexes were explored to provide cost-effective bioindicators for pollution monitoring and mitigation.

**Abstract:**

This research investigates the dynamics of contaminant exposure in European hake (*Merluccius merluccius*, L. 1758) from the Adriatic Sea (Central Mediterranean Sea) by examining the levels of total mercury (THg) and polycyclic aromatic hydrocarbons (PAHs) in the muscle fish tissues. The study explores the correlations between these pollutants and somatic indexes to identify the early warning signals of pollution and ecological effects. The levels of pollutants are influenced by season and sex. Lipids appear to have a minimal effect on the PAH levels, whereas they exhibit a positive correlation with mercury levels in the muscle. No significant relationships between the pollutants and condition indexes were observed, except for a positive correlation between THg and the gonadosomatic index, indicating a potential impact on the reproductive health of fish. In contrast, PAHs showed no meaningful correlation with condition indexes. Differences in contaminant accumulations and lipid levels between sexes reflect variations in metabolic activity, reproductive costs, and adaptive strategies to seasonal changes and energy demands. This study highlights the importance of long-term monitoring to improve pollution management, environmental conservation, and the protection of marine organisms’ health.

## 1. Introduction

In recent years, there has been increasing awareness of the significant influence of pollutants on the marine ecosystems within the Mediterranean basin, particularly affecting demersal and pelagic fishery resources. This partially enclosed sea, known for its limited water circulation, is one of the most heavily polluted bodies of water globally [1]. These resources are already under significant pressure due to overfishing, habitat degradation, and not uniformly efficient management practices [2,3,4,5,6,7].

This concern has prompted extensive research into the diverse effects of pollutants, focusing on the long-term preservation of marine resources [8,9,10].

Aquatic organisms, including fish, accumulate pollutants both directly from contaminated water and indirectly through the food web [7,11,12]. The effects of pollutants on aquatic organisms vary based on the toxicity and concentration of the contaminants [11], with significant variability among individuals, species, and life stages [13,14,15]. These effects can influence organ functions, reproductive status, and population size, determined by the species’ tolerance and survival capabilities [16].

Fish are notable for their ability to metabolize, concentrate, and retain pollutants, making them important bioindicators for evaluating environmental stress and detecting toxicant-induced alterations and degradations over time and space [11,12,17,18,19,20]. They also offer insights into human exposure risks through the aquatic food chain [21,22,23,24,25,26]. Consequently, monitoring pollutant levels in marine organisms is crucial to ensure environmental safety by establishing legislative limits.

The Adriatic Sea, a productive fishing ground and integral part of the Mediterranean, is vulnerable to environmental pollution from human activities like resource exploitation, agricultural runoff, coastal urban development, heavy shipping traffic, and substantial inputs from industrialized Italian rivers [7,27,28,29,30]. Its unique oceanographic and geographical characteristics enhance contaminant biomagnification in organisms [31,32,33,34]. For these reasons, it is urgent to elucidate the pollution status in the Adriatic Sea, focusing on the accumulation and persistence of contaminants like trace elements and PAHs in the environment and marine organisms, especially in edible fishes.

However, measuring xenobiotic concentrations and implementing monitoring programs can be costly and may not fully reflect their impact on biological systems [35,36]. Moreover, chronic exposure to even low levels of toxic elements can have adverse effects on ecosystems which is often not fully investigated [37,38].

Recent advances in ecotoxicology have extensively employed bioindicators and condition indexes in fish populations as efficient early warning tools for assessing chemical exposure impacts on environmental quality [39,40,41,42,43,44,45]. These indexes can help initiate bioremediation strategies before irreversible environmental damage occurs.

Somatic indexes, such as the gonadosomatic index (GSI), hepatosomatic index (HSI), and the Le Cren condition factor (Kn), can be indicative of fish health and describe physiological changes due to pollutant exposure and environmental stressors [46,47,48]. The GSI provides information about gonadal health and maturation, with significant evidence showing that pollutant exposure can reduce GSI and cause morphological changes in gonads [49,50]. The HSI is a well-known bioindicator of contaminant exposure, as the liver plays a crucial role in detoxification, with pollutants causing an increase in liver size due to hypertrophy (enlargement) or hyperplasia (increased number of liver cells), or both [51,52,53]. The Kn [54]) is a quantitative indicator of individual well-being, reflecting recent food availability and energy reserves [55,56].

This study investigates GSI, HSI, and Kn in European hake (*Merluccius merluccius*, L. 1758) from the Adriatic Sea, relating them to the levels of mercury (Hg) and polycyclic aromatic hydrocarbon (PAHs) levels recorded in the fish muscle. This approach aims to gain a deeper understanding of the environmental and biological factors influencing pollutant accumulation and provide insights into the physiological responses of European hake to environmental stressors, potentially revealing simple, cost-effective bioindicators suitable for routine pollution monitoring and mitigation for this important species in the Adriatic Sea.

This species is widely distributed across the Atlantic and the Mediterranean and Black Sea and supports important fisheries in several Mediterranean regions, making it a key species for both economic and ecological reasons. However, it has faced significant overfishing, although in recent decades, its critical overexploitation condition has slowly improved, with the landings in the Mediterranean decreasing from 52,394 tonnes in 1994 to 17,824 tonnes in 2021 [57]. In 2022, hake landings in the Mediterranean Sea totaled 18,388 tons with 14% from the northern and central Adriatic [58]. The risk of stock collapse has been highlighted by several scientists [6,59,60,61], underscoring the need for comprehensive studies on the impacts of pollutants on this species and the broader ecosystem.

Mercury and PAHs exhibit distinct physicochemical properties and interaction patterns that influence their toxicity. Both contaminants partially metabolize and accumulate in lipid-rich tissues like fish muscle, posing potential risks to human health through consumption [62]. Recent research shows that PAH mixtures induce cellular stress and metabolic disruptions in fish, leading to oxidative stress, endocrine issues, and development problems, which affect their survival, growth, and reproduction [9,63,64,65].

Mercury, even in low concentrations, can damage cells, tissues, proteins, and genes; disrupt physiological processes; and impair behavior and reproductive health [66,67,68,69,70]. It can also trigger energy-consuming detoxification processes, reducing the energy available for growth [70].

The data on Hg levels have already been previously analyzed [71], but in this study, we have expanded on those analyses by examining the relationship between the Hg and PAH levels observed in the muscle tissue of *Merluccius merluccius* and various somatic indexes, such as GSI, HSI, and Kn, to identify the early warning signals of pollution and ecological effects.

## 2. Materials and Methods

### 2.1. Study Area and Fish Sampling

**Figure 1:**
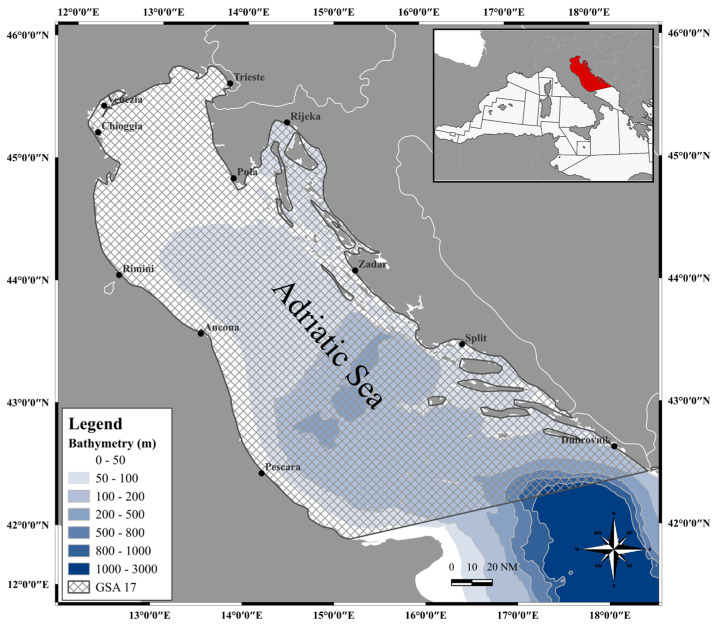
Map of the study area GSA 17, elaborated by I. Costantini (software: QGIS Development Team, 2024. QGIS Geographic Information System. Open Source Geospatial Foundation Project. http://qgis.osgeo.org, accessed on 30 May 2024).

European hake (*Merluccius merluccius*) individuals (*n* = 163) were seasonally sampled by commercial bottom trawlers from May 2018 to January 2019 in a productive offshore fishing ground spanning the Northern and Central Adriatic Sea (FAO-GFCM geographical sub-area 17, Figure 1).

In the laboratory, each individual’s body length was measured with a precision of 0.5 cm, total weight was recorded to 0.1 g (Radwag WLC–6F1/K precision scale, Radwang Wagi Elektroniczne, Radom, Poland), and sex was determined [72].

The sampling procedures did not include any animal experimentation and animal ethics approval was, therefore, not necessary under the Italian legislation (D.L. 4 March 2014, n. 26, art. 2). The methodology and sampling of this study were based on dead specimens from professional fishing.

The gonads and liver were weighed to the nearest 0.001 g (Mettler Toledo XP204, analytical precision scale) in order to calculate the somatic indexes, GSI, HSI, and Kn, using the following equations:

GSI (%) = gonad weight/GW body × 100

HSI (%) = liver weight/GW body × 100

Kn = W/*a* TL*^b^*

where *a* and *b* were the regression parameters of the length-weight relationship, GW was the gutted weight, and TL was the total length.

### 2.2. Laboratory Analyses and Total Mercury Determination

All the analytical procedures were conducted in a clean room laboratory conforming to the ISO 14644-1 Class 6 standards, with certain areas meeting ISO Class 5 requirements under laminar flow. Prior to use, all the laboratory equipment underwent a rigorous acid-cleaning procedure [73]. The specimens were weighed using an AT261 analytical balance (Mettler Toledo, AG Laboratory & Weighing Technologies, Greifensee, Switzerland), readability 0.01 mg, repeatability standard deviation 0.015 mg). Different fish tissues, such as the gonads, liver, and muscle, were dissected using a scalpel that had been previously cleaned with acetone to avoid contaminating the sample.

Variable volume micropipettes and neutral tips from Brand (Transferpette, Wertheim, Germany) and scalpels with sterile stainless-steel blades from Granton (Mod. 91021, Sheffield, UK) were employed. Ultrapure water was obtained from a Milli–Q water system (Merck Millipore in Darmstadt, Germany). The acetone and petroleum ether utilized for lipid extraction were RS grade for pesticide analysis (Carlo Erba, Milano, Italy). Certified reference material for Hg content, specifically dogfish muscle DORM-2 (NRCC, Ottawa, ON, Canada), was utilized.

For Hg determination in fish muscle, approximately 0.05 g of each sample, which was minced and homogenized (homogenizer MZ 4110, DCG Eltronic, Monza, Italy), was directly analyzed via thermal decomposition amalgamation atomic absorption spectrometry (TDA AAS) utilizing a Direct Mercury Analyzer (DMA-1, FKV, Milestone, Sorisole, Italy) [74,75,76,77]. The sequential steps followed the protocol indicated by Annibaldi et al., [78]. Detection cell ranges were set between 0.03–200 ng and 200–1500 ng.

Mercury concentrations were quantified using the calibration curve technique. For each specimen, the analysis was performed in triplicate and the THg concentration of a blank was subtracted from the sample measurements to account for potential mercury contamination during the analysis. The use of DORM–2 certified reference material ensured analytical quality control. The mean experimental THg value of 4.47 ± 0.10 mg kg^−1^ wet weight (ww), closely matching (*p* > 0.05) the certified values (4.43 ± 0.05 mg kg^−1^ ww), indicated method accuracy and repeatability.

### 2.3. Polycyclic Aromatic Hydrocarbon (PAH) Extraction and Analysis

The Quick Easy Cheap Effective Rugged and Safe (QuEChER) method was employed to extract and purify PAHs from the muscle of *M. merluccius*. This method represents a simple, rapid, and cost-effective alternative to the conventional extraction methods for multi-residue analysis [79]. By requiring only a few steps (extraction and clean-up), it reduces both time and solvent consumption. The QuEChERS method, originally designed for pesticides, underwent modifications to other persistent organic pollutants, such as PAHs [80].

For PAH analysis, a portion of approximately 5 g of muscle was taken dorsally, directly under the anterior dorsal fin and well above the lateral line, using solvent-cleaned scalpels and scissors. Each sample was wrapped in aluminum foil and stored in a freezer at −18 °C.

The extraction of PAHs using the QuEChERS method was conducted using some kits for the partition of the compounds from the aqueous solution and acetonitrile as solvent extraction. This partitioning process involved the addition of MgSO_4_ and NaCl. Subsequently, the samples underwent purification through dispersive solid-phase extraction (dSPE) clean-up using C18, MgSO_4_, and primary secondary amine (PSA). The purified extracts were then evaporated under a gentle flow of N_2_ and finally recovered with acetonitrile for chemical analysis. The analysis was conducted using UHPLC (Ultimate 3000, Thermo Scientific, Waltham, MA, USA), which was equipped with a fluorescence detector (RF2000, Thermo Scientific). For separation, a Hypersil Green PAH column (2.1 × 150 mm, 1.8 μm, 120 Å) in a reversed phase was used.

Total PAHs include the sum of the sixteen most environmentally relevant PAHs, which are naphthalene, acenaphthylene, acenaphthene, fluorene, phenanthrene, anthracene, fluoranthene, pyrene, benz(a)anthracene, chrysene, benzo(b)fluoranthene, benzo(k)fluoranthene, benz(a)pyrene, dibenz(a,h)anthracene, benzo(ghi)perylene, and indeno(1,2,3–c,d)pyrene, listed in the US Environmental Protection Agency (EPA) priority pollutant list.

The analysis for quality control assessment involved both the use of an external standard multipoint calibration technique and procedural blanks (*n* = 6). Calibration curves were performed via serial dilutions ranging from 1:400 to 1:3200 *v*/*v* of a standard PAH solution of EPA 610 PAH Mix (Supelco, Bellafonte, PA, USA). The mean recovery rate was calculated at 87% (±5%) and no correction for surrogate recoveries was applied. The limits of detection (LOD) and quantification (LOQ) were calculated following the ICH Q2B guidelines [81] (ICH, 2005), resulting in LODs ranging from 0.001 to 0.01 ppb and LOQs ranging from 0.004 to 0.4 ppb. The concentrations of all the PAH compounds in the blank extract samples remained below the LOQ.

### 2.4. Lipid Content in Fish Muscle

The muscle tissue from each sample specimen underwent mincing, homogenization, precise weighing, and freeze-drying using an Edwards EF4 Modulyo freeze dryer in Crawley, Sussex, UK, until reaching a constant weight (±0.2 mg). The total lipid content was determined in triplicate aliquots from each specimen and the average moisture percentage was calculated.

Microwave-Assisted Extraction (MAE) involved placing 0.5 g of each portion into a Teflon extraction vessel with 10 mL petroleum ether and 5 mL acetone in a Microwave–Accelerated Reaction System (MARS-5, 1500 W; CEM, Mathews, NC, USA; [82,83,84].

The resulting extract underwent filtration through Whatman GF/C filter papers (Ø 90 mm, GE Healthcare Life Sciences, Buckinghamshire, UK) containing anhydrous sodium sulfate (Carlo Erba) and was rinsed twice with an additional 2 mL of a petroleum ether-acetone mixture (2:1 *v*/*v*). Subsequently, the filtrate was evaporated under laminar flow inert gas (N_2_) until reaching a constant weight, after which the mass of the extracted lipids was determined.

### 2.5. Statistical Analysis

The dataset consisted of 163 observations and eight variables. Among these, two were factor variables: season (with four levels: spring, summer, autumn, and winter) and sex (with two levels: male (M) and female (F)). The remaining six were numeric variables: total mercury, total PAHs, lipids, HSI, GSI, and CF.

Due to the limited availability of muscle samples, it was not feasible to test for both contaminants (PAHs and Hg) in all individuals. Out of 163 samples, Hg was analyzed in 74 individuals, PAHs in 151 individuals, and both contaminants were analyzed together in only 51 individuals.

The Season levels were categorized according to Artegiani et al., (1997a,b) [32,33] as follows: spring: April–June; summer: July–September; autumn: October–December; and winter: January–March.

There were some randomly distributed missing values in the dataset: 89 for total mercury, 12 for total PAHs, 21 for lipids, 25 for GSI, 34 for HSI, and 3 for Kn. However, in each of the statistical analyses, only the individuals with a full record (no missing value) were considered.

A 4 × 2 Analysis of Variance (ANOVA) design with interactions was employed to explore the effects of the two independent variables (season and sex) on the dependent variables (total mercury, total PAHs, lipids, HSI, GSI, and Kn). However, the limited number of observations and the stratification for the factor levels reduced the amount of data per cell, sometimes less than 20, posing challenges for assessing the normality distribution of the data [85]. In order to overcome the problems of normality distribution and homogeneity of variances, we directly applied a nonparametric ANOVA, based on the Aligned Rank Transform (ART), with the corresponding post hoc comparisons [86,87]. For the nonparametric two-way ANOVA, we used the function art from the R package ARTool. The function art.con from the same package was used for the post hoc comparisons. A reference *p* value of 0.05 was considered for significance. For the post hoc tests, the default Tukey HSD correction for multiple comparisons was applied [88].

For the post hoc comparisons, in the discussion of the results we focused on the main results, which include the differences among the seasons for each sex and the differences between the sexes in each season. As described above, the omnibus ANOVA and post hoc comparisons are based on the Aligned Rank Transform (ART). However, when discussing the results from a biological perspective, medians were referenced for the variables of interest since the median is more meaningful than the mean in the case of skewed distributions, as observed in our data.

Due to the limited number of data, which prevented assessing the normality assumption (see above), the correlation coefficients among the numeric variables were calculated using the nonparametric Kendall’s tau, which has fewer assumptions than the Pearson coefficient [89]. Initially, this analysis was conducted without stratification for season and sex; subsequently, the data were stratified for season and sex and Kendall’s tau was recalculated. Due to the numerous pairwise tests, the usual *p* value for significance (0.05) was adjusted using Bonferroni’s correction [90] for the number of tests (15), resulting in an adjusted significance level of 0.003 (=0.05/15).

All the statistical analyses were performed using the free statistical software R ver 4.4.0 [91].

## 3. Results

### 3.1. Total Mercury Levels in Fish Muscle

The study estimated the total mercury level in the muscle tissue of 74 European hake individuals (38 females and 36 males) considering seasonal variations and sex differences.

The total mercury concentration (THg) ranged from 0.03 to 0.37 mg kg^−1^ ww (Table 1). Detailed data on the THg levels and body size of *Merluccius merluccius* are presented in Table S1 in Supplementary Materials. The omnibus ANOVA results and the pairwise post hoc comparisons are reported in Supplementary Materials (Table S2).

The results of the omnibus two-way ANOVA with interaction indicated that there is a marked effect of season (F (3, 66) = 5.625, *p* value = 0.002), no effect of sex (F(1, 66) = 0.095, *p* = 0.759), and a weak effect of interaction (F (3, 66) = 3.124, *p* = 0.032; Table S2).

**Figure 2:**
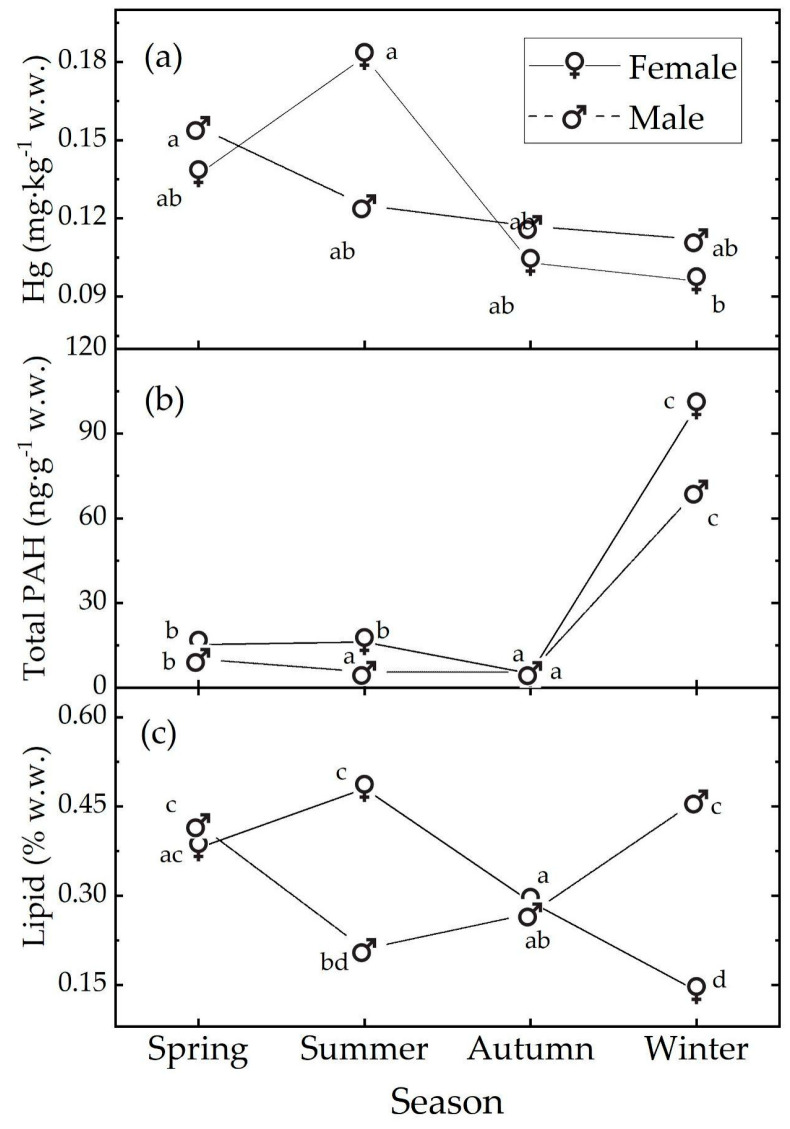
Median levels of (**a**) Hg, (**b**) total PAH, and (**c**) % lipids observed in the muscle tissue of *M. merluccius* as a function of sex and season. The medians with different letters differ at the *p* value = 0.05. The letters refer to the post hoc ART results; see text and Supplementary Materials.

In the following, we will discuss only the main post hoc results. In females, the Hg concentration exhibited a seasonal trend from a peak in summer to a minimum in winter (Table 1 and Figure 2a). The value in summer is almost double that in winter and this difference is strongly significant (t.ratio(66) = 4.465, *p* value < 0.001). In males, a seasonal trend is not evident, and the data are compatible with the null hypothesis of no season influence. In each season, the data are compatible with the null hypothesis of no sex influence, see Table S2.

### 3.2. Total PAH Levels in Fish Muscle

The study also examined the total levels of polycyclic aromatic hydrocarbons (PAHs) in 151 individuals (75 females and 76 males), with concentrations ranging from 2.70 to 185.79 ng g^−1^ wet weight (Table 2). Detailed PAH concentrations are provided in Table S3 in Supplementary Materials.

The results of the omnibus two-way ANOVA with interaction indicated that there is a strong effect of season (F (3, 143) = 90.573, *p* < 0.001), sex (F (1, 143) = 50.798, *p* < 0.001), and their interaction (F (3, 143) = 6.336, *p* < 0.001). These results, and the pairwise post hoc comparisons, are reported in Table S4 in Supplementary Materials.

In females, the total PAH levels exhibited a clear seasonal trend: they remained almost stable during spring and summer, declined in autumn to the lowest value, and peaked in winter (Table 2 and Figure 2b). Males showed a similar pattern, with the lowest PAH levels in autumn and the highest in winter; though in this case, the summer levels were closer to those in autumn than in spring. During spring, autumn, and winter, our data are compatible with the null hypothesis of no sex influence. In contrast, in summer, the data suggest a sex influence, with the female samples showing higher pollution levels than the male samples (t.ratio (143) = 7.093, *p* value < 0.001; Table S4).

### 3.3. Lipid Levels in Fish Muscle

Considering the propensity for PAHs and Hg to accumulate in lipids, the study estimated the lipid levels in the muscle of 142 European hake individuals (66 females and 76 males), taking into account seasonal variations and sex differences. The total lipid levels, expressed as % wet weight (w.w.), ranged from 0.14 to 0.52 (Figure 2c).

The results of the omnibus two-way ANOVA with interaction indicated that there is a strong effect of season (F (3, 134) = 56.796, *p* < 0.001), sex (F (1, 134) = 105.164, *p* < 0.001), and their interaction (F (3, 134) = 113.647, *p* < 0.001). These results, and the pairwise post hoc comparisons, are reported in Table S5 in Supplementary Materials.

In females, lipids exhibited a clear seasonal trend, increasing from spring, reaching the maximum in summer, and decreasing to a minimum value in winter, indicating the lowest lipid reserves during this season (Figure 2c). Males also showed a seasonal pattern, but their peak levels were observed in winter, with the minimum in summer. In spring and autumn, our data are compatible with the null hypothesis of no sex influence on lipid levels (Table S5). However, in summer and winter, our data suggest a sex influence: in summer, the female samples had higher lipid levels than the male samples (t.ratio (134) = 8.197, *p* < 0.001), while the opposite was observed in winter (t.ratio (134) = −10.832, *p* < 0.001).

### 3.4. Somatic Indexes: GSI, HSI, and Le Cren Kn

#### 3.4.1. Gonadosomatic Index (GSI)

The study estimated the GSI in 138 European hake individuals (69 females and 69 males) considering seasonal variations and sex differences. The GSI ranged from 0.05 to 11.23 (Table 3).

The results of the omnibus two-way ANOVA with interaction indicate a strong effect of season, (F (3, 130) = 10.599, *p* < 0.001), sex (F (1, 130) = 31.193, *p* < 0.001), and their interaction (F (3, 130) = 13.369, *p* < 0.001). Detailed results and the pairwise post hoc comparisons are available in Table S6 in Supplementary Materials.

**Figure 3:**
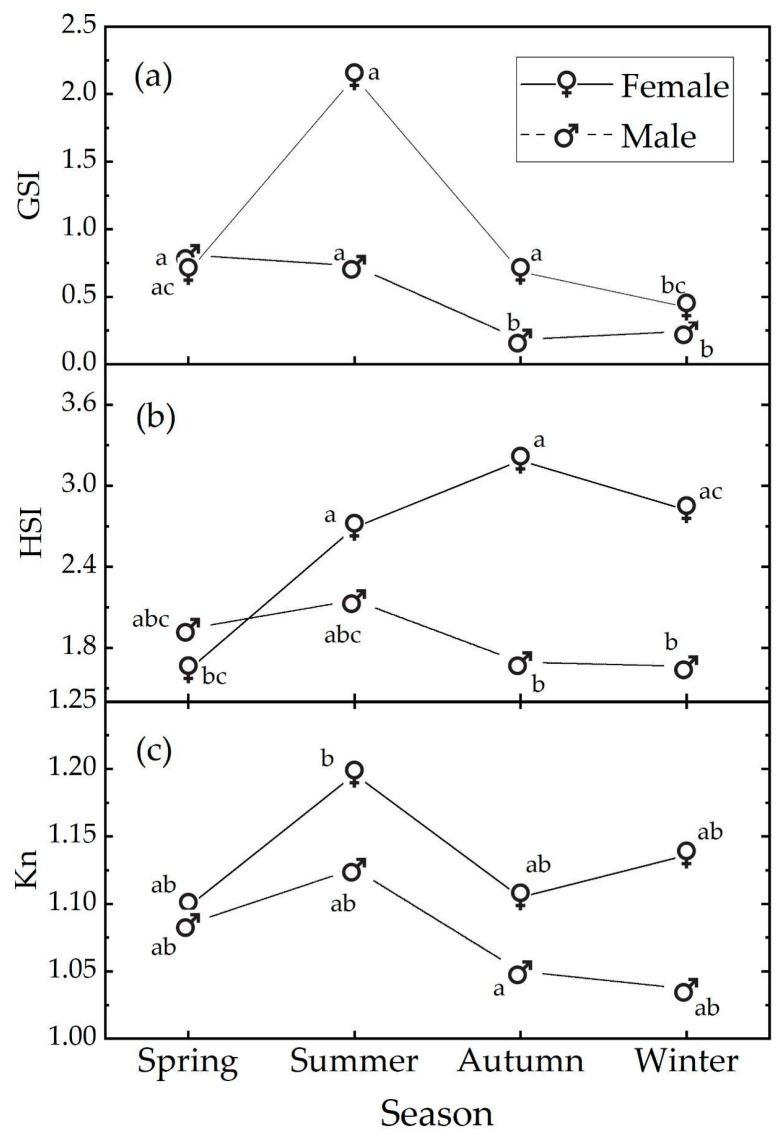
Median values of (**a**) GSI, (**b**) HSI, and (**c**) Kn Le Cren indexes as a function of sex and season. Medians with different letters differ at the *p* value = 0.05. The letters refer to the post hoc ART results; see text and Supplementary Materials.

In females, the GSI reached its highest value in the summer, corresponding to the maximum gonadal development during the spawning peak (Figure 3a). The GSI then gradually decreased in autumn (October–December), returning to the value observed in spring, and reached its lowest in winter, indicating that more individuals were recorded in the post-reproductive gonadal maturation condition, although European hake is a protracted reproductive season species [92].

Males also exhibited a seasonal pattern, but the peak value was observed in spring/summer, with the minimum in autumn/winter [93].

In winter, spring, and summer, our data are compatible with the null hypothesis of no sex influence on GSI levels. However, in autumn, our data suggest a sex influence, with the female samples showing higher GSI levels than the male samples (t.ratio (130) = 5.095; *p* < 0.001).

#### 3.4.2. Hepatosomatic Index (HSI)

HSI was estimated in 129 individuals of European hake (59 females and 70 males), taking into account seasonal variations and sex differences. The HSI level ranged from 0.86 to 5.69. Detailed data are reported in Table 4.

The results of the omnibus two-way ANOVA with interaction indicated that there is no effect of the season (F (3, 121) = 1.041, *p* = 0.377), and a strong effect of sex (F (1, 121) = 10.776, *p* = 0.001) and their interaction (F (3, 121) = 5.505, *p* = 0.001). These results and the pairwise post hoc comparisons are reported in Table S7 in Supplementary Materials.

In females, the HSI showed a mild seasonal trend, gradually increasing from its lowest value in spring to its highest in autumn, followed by a slight decrease in winter. Males exhibited no statistical seasonal pattern, with the highest HSI value occurring in summer and the lowest in winter (Figure 3b). The data indicated a sex influence only in autumn with females having a higher HSI level than males (t.ratio (121) = 3.497, *p* = 0.015).

#### 3.4.3. Le Cren’s Kn

Le Cren’s Kn was calculated in 160 individuals (85 females and 75 males) considering seasonal variations and sex differences. This index ranged from 0.87 to 1.48 (Table 5).

The omnibus two-way ANOVA with interaction showed no season effect (F (3, 152) = 2.528, *p* = 0.059), a weak sex effect (F (1, 152) = 5.760, *p* = 0.018), and no interaction effect (F (3, 152) = 1.121, *p* value = 0.342). Detailed results are available in Table S8 in Supplementary Materials.

Regarding Le Cren’s Kn index, similar values were observed across all the seasons for both sexes (Figure 3c). The post hoc comparisons indicate that the Kn index data are substantially compatible with the null hypothesis of no influence of season or sex; i.e., despite the significant sex effect in the omnibus ANOVA test, none of the relative post hoc comparisons were significant (Table S8). This is due to the higher power of the omnibus ANOVA test according to Freund et al. (2010) [94].

### 3.5. Correlations between Pollutants, Lipids, and Somatic Indexes

A comprehensive statistical analysis was conducted to examine the relationships between the pollutants, lipids recorded in the muscle of European hake, and various somatic indexes. Despite the thorough investigation, only a few of these relationships were found to be statistically significant. The detailed results are summarized in Table 6, which provides a summary of the pairwise Kendall’s tau correlations for the numerical variables. Kendall’s tau is a measure of correlation that assesses the strength and direction of the association between two variables. This nonparametric statistic is particularly useful for understanding the relationships in ordinal data or when the data do not meet the assumptions of parametric tests.

Initially, this analysis was conducted on the entire dataset without stratification for season and sex; subsequently, the data were stratified for season and sex and Kendall’s tau was recalculated (see Tables S9–S16 in Supplementary Materials).

Throughout the year, a significant positive relationship between the total Hg and the GSI was observed for both sexes combined, with no noticeable seasonal variations. In contrast, no significant relationship was detected between the total PAHs and GSI.

Additionally, a positive relationship between the Hg and lipid content was noted, although this correlation was not significant within individual seasons. For PAHs, there was no significant relationship observed.

A generally positive correlation between the lipid content and GSI was observed for both sexes combined throughout the year. This trend was not so evident when examining individual seasons and single-sex groups. This correlation is crucial, as lipid accumulation is vital for the accumulation of reserves in preparation for gamete development and spawning (see Tables S9–S16 in Supplementary Materials and values for detailed data).

Furthermore, a positive correlation was noted between GSI and HSI, as well as between HSI and Le Cren’s Kn. Interestingly, a negative relationship between PAHs and Hg was observed throughout the year for both sexes combined, although this was not statistically significant. This result suggests that as the PAH levels increase, the Hg levels tend to decrease, and vice versa. Overall, no significant seasonal patterns were evident.

## 4. Discussion

The accumulations of contaminants in muscle tissue are influenced by various abiotic (water, sediment, and geographic location), biotic (size, sex, age, and reproduction stage), and ecological factors like growth rate, feeding sources, and trophic level [13,14,18,95,96,97].

These factors, individually or collectively, can cause significant stress, leading to reduced growth, impaired reproduction, increased disease susceptibility, and decreased ability to tolerate further stress. At the population level, stress effects can result in reduced recruitment and compensatory reserve.

Toxicants can affect organisms at various biological levels, making it inadequate to rely on a single stress response for a comprehensive assessment. The bioindicator approach, which evaluates multiple responses, effectively measures sublethal stress effects on fish. This approach serves as an early warning system and could help clarify the relationships between stressors and their broader biological impacts.

Bioindicators and condition indexes provide a comprehensive view of fish health and environmental stress, reducing misinterpretation and offering cost-effective pollution monitoring tools essential for evaluating long-term contaminant impacts.

This study applies the bioindicator approach to assess stress responses in relation to European hake (*Merluccius merluccius*), one of the most important commercial species fished in the Adriatic Sea. The fish were analyzed specifically for Hg and PAH levels in their muscle tissue and condition indexes.

This species, characterized by indeterminate fecundity [98,99,100], a protracted spawning season [101], and a complex reproductive strategy [102], is particularly vulnerable to the negative individual- and population-level impacts of chronic contaminant exposure.

In the Mediterranean, European hake typically spawns in spring-summer, peaking from April to July, though this timing can vary depending on the location and environmental conditions [103,104,105,106,107,108].

Both sexes experience metabolic and hormonal changes [109] during reproductive stages, affecting pollutant accumulation in muscle tissue.

This study found Hg levels in muscle tissue (0.03 to 0.37 mg kg^−1^ dw) generally consistent with those reported in the Adriatic Sea [110,111] and other parts of the Mediterranean Sea [112,113,114]. On the contrary, Jureša and Blanuša (2003) [115] and Perugini et al. (2014) [116] observed values well higher than these in the Adriatic Sea.

No significant differences in the THg levels were observed between the sexes, but females exhibited important seasonal variations, with peak levels in summer and lower levels in winter. In contrast, males maintained stable THg levels throughout the year (Figure 2a).

Several studies have shown that the Hg levels in the edible tissues of females decrease after spawning [117,118,119,120]. This reduction in Hg levels may be due to a sort of “cleaning process” where contaminants are eliminated during oocyte release [121,122,123].

This pattern is less evident in males, whose lipid content and energy reserves did not change greatly during the year. This is likely due to continuous spermatogenesis in adult specimens without a significant resting phase [93,124].

In most of the muscle samples analyzed, the total PAH levels recorded in this study fell within the “minimally polluted” category (10–99 ng/g), with a mean concentration of 33.1 ± 40.6 ng/g ww [125]. The total PAH concentrations detected in this study are comparable to those reported in the study by [126] on European hake caught in the Gulf of Taranto during the period from February to July 2010. However, the values found in the *M. merluccius* specimens from the Adriatic Sea examined in this study show lower concentrations compared to those sampled in the Gulf of Taranto (40–350 ng/g ww). Conversely, the PAH levels in the present study are higher than those found in the hake sampled in the Gulf of Naples in the study by [127] which reported the average total PAH values of 6 ng/g ww, and those reported in the study by [128], whose total PAH concentrations in the muscle of *M. merluccius* ranged from 3 to 7 ng/g ww. Meanwhile, the levels detected in the present study are very similar to those reported by [127] for samples from the Adriatic Sea, where the average total PAH concentrations in *M. merluccius* were about 44 ng/g.

Higher levels of total PAH concentration were recorded during the winter season compared to the summer, for both the male and female individuals (Table S2, Figure 2b). This can be attributed to both anthropogenic and natural factors. In the colder months, there is indeed a higher release of these substances into the atmosphere compared to the summer months due to the increased use of domestic heating systems as well as greater vehicular traffic during winter. In the marine environment, the winter months are associated with increased riverine discharge due to elevated levels of atmospheric precipitation. Additionally, lower winter temperatures inhibit all the PAH biodegradation processes performed by microorganisms, affecting the solubility, dispersion, and bioavailability of these contaminants. During the winter period, the reduced solar radiation intensity could decrease the efficiency of PAH photodegradation, leading to the increased persistence of these compounds in the marine environment, particularly within marine sediments. Additionally, marine sediments are subjected to remixing and bioturbation phenomena during the winter period, which can disrupt the equilibrium at the water-sediment interface and facilitate the release of previously deposited PAHs.

However, the samples were derived from commercial fishing operations and did not include the full range of upper size and age classes. As a result, the study did not investigate the relationship between contaminant levels and fish size, potentially missing important variations in contaminant distribution within the population. Future research with a larger sample size could provide more detailed insight and improve the reliability of the contamination assessment. Lipids, while serving as energy reserves, are considered crucial for the accumulation of pollutants like polycyclic aromatic hydrocarbons (PAHs) and mercury (Hg). Due to their hydrophobic nature, these compounds tend to associate with lipid-rich tissues in organisms. These pollutants can exert toxic effects and be transferred across trophic levels, affecting ecosystems [11,18,129,130,131].

In our study, lipids seem to exert a limited influence on the PAH levels in the muscle of European hake while showing a strong positive correlation with Hg. Lipid distributions in muscle tissue exhibited distinct seasonal trends and differences between sexes, displaying notable changes during summer and winter.

In females, the lipid levels were highest from spring to summer, likely due to energy storage for reproduction and oocyte development, and then decreased to their lowest reserves in winter after reproductive effort and egg release.

In contrast, males exhibited less variation in lipid content throughout the year, although they showed distinct seasonal trends, possibly due to continuous spermatogenesis, with the “resting phase” almost absent [93,124]. Males’ lipid levels were highest in winter, aligning with pre-spawning energy reserves.

These differences in lipid level reflect variations in metabolic activity, costs of reproduction, and adaptive strategies of both sexes to seasonal environmental changes and energy demands.

Previous studies have shown extreme variability in correlations between lipid content and organic contaminants. Some reported positive correlations between PAH accumulation and lipids [132,133,134], while others found weak [131], negative [135], or no correlations [136,137].

Similarly, the importance of lipids in relation to Hg levels is quite contradictory [120,138,139].

This inconsistency suggests that lipid content may not be the key factor for tissue-specific pollutant accumulation in fish, indicating the variability of this relationship among different fish species, environmental conditions, and especially trophic habits [140,141,142,143,144].

Furthermore, high variation in lipid reserves between individuals of the same species is common in wild fish [145] and may reflect significant differences in nutritional status and reproductive potential within a population. This strong inter-population variability likely explains the low correlation values observed between the different variables studied.

Somatic indexes like the GSI, HSI, and Kn were valuable for assessing pollutant impacts on aquatic ecosystems. These indexes are sensitive indicators of changes in fish physiology, allowing researchers to monitor the subtle effects of pollutants on fish populations. Extensive research supports their use as reliable proxies for assessing fish responses to environmental stressors [46,47,48].

A comprehensive analysis conducted throughout the year revealed no relationships between the two pollutants and condition indexes, except for a positive correlation between the total Hg and the GSI, without significant seasonal and sex variations. In contrast, PAHs showed no meaningful correlation with condition indexes. This could be due to the fact that the study focused on wild fish rather than those exposed to controlled levels of contamination, where the effects of the contaminants on these indexes would likely be more directly measurable.

A negative but not statistically significant relationship between PAHs and Hg was observed, suggesting that as PAH levels increase, Hg levels tend to decrease, and vice versa with no significant seasonal patterns.

Conversely, significant relationships were found among the condition indexes, highlighting energy intake utilization. Specifically, a positive correlation was noted between GSI and HSI, as well as between HSI and Kn.

To understand the reproductive strategy in terms of energy investment, assessing the seasonal variability of somatic and gonadic conditions is essential. Fish alternated their energy use between body growth and reserves (fat, muscle, and liver) and gonadic growth throughout the year.

In spring and summer, females allocated energy to reproduction both from concurrent feeding [93,107,146] and from lipid reserves to support a long reproductive cycle; in these seasons, the GSI increased progressively from April to August before sharply decreasing in autumn. This pattern indicates energy allocation towards reproductive efforts.

Males exhibited low GSI with minimal seasonal variation, showing less energy investment in gonad development. In autumn, the GSI for both sexes decreased, reaching its lowest point in winter, signifying post-spawning recovery [92].

The HSI was generally higher in females and remained constant in males throughout the year. Le Cren’s Kn showed similar trends for both sexes, with females consistently having slightly higher values. Seasonal variability in body condition, in terms of weight and length, was more pronounced in females, suggesting an increase in body size from spring to summer, followed by a decline in the condition thereafter.

In spring and summer, during the preparation for spawning, females’ lipid content and Kn values increased due to the accumulation of muscle and lipid reserves. Although gonad production increased during this period, the maintenance of body condition and liver mass growth suggests that external energy sources, such as food, supported both gonadic growth and body mass maintenance. This indicates that energy intake during the spawning season plays a crucial role in growth capacity, rather than relying solely on energy reserves accumulated several months prior. Similar to females, males used muscle lipids as energy reserves during this period.

In autumn, during the post-spawning period, the female hake exhibited an increase in HSI and a decrease in the lipid content and Kn, indicating the use of muscle lipids and proteins as energy reserves. Despite the increase in gonad production, stable body condition levels and liver mass growth suggest ongoing external energy support.

Males showed a decrease in Kn, but an increase in the lipid content, indicating reserve accumulation.

In winter, females showed a decrease in HSI and an increase in Kn, with a decline in the lipid content, reflecting the use of lipids as fuel during food shortages, almost depleting muscle lipids. Males, however, exhibited a decrease in Kn and an increase in the lipid content, continuing to accumulate reserves.

In Adriatic waters, high Kn and HSI levels from summer to winter indicate ample feeding resources for female somatic and gonadic growth. After the main spawning peak, a decrease in GSI and an increase in HSI suggest that adults do not end the spawning season exhausted. Hake continue feeding during the breeding season, unlike in cold waters where spawning occurs in winter with scarce food, and vitellogenesis relies on stored energy from previous months [100].

These observations highlight seasonal differences in energy use between sexes, influenced by environmental conditions and food availability.

## 5. Conclusions

The study revealed that the contaminant levels in European hake (*Merluccius merluccius*) from the Adriatic are below international safety limits. However, regular monitoring is essential for maintaining the health of marine ecosystems. The research also emphasizes the importance of understanding and mitigating the impact of pollutants on marine organisms’ reproductive health to protect marine ecosystems worldwide.

A complex interaction between the PAHs and Hg levels in muscle tissues is mainly influenced by both sex and seasonal factors. PAH levels are more affected by seasonal changes with higher concentrations in the winter season, while Hg levels in females are consistently associated with lipid content throughout the year.

No significant correlation was found between PAHs and gonadosomatic or morphometric indexes, likely because this study focused on wild fish rather than those exposed to controlled contamination, where contaminant effects would be more measurable. Seasonal fluctuations in contaminant levels seem to be chiefly related to metabolic activity, the reproductive cycle, and the energy costs in terms of the energy of reproduction, growth, and physiological processes being different for the sexes, affecting tissue composition in terms of lipid content. This variation affects the concentration of the investigated contaminants, suggesting that fish physiological conditions should be considered in biomonitoring programs.

During the breeding season, part of the assimilated energy is allocated to gamete production, reducing dependence on energy reserves. Females feed during breeding, indicating that liver lipids stored during maturation are mobilized towards the gonads for reproductive purposes.

Future studies are planned to clarify how lipid dynamics and seasonal and sex-based variations in lipid levels influence the deposition of mercury (Hg) and polycyclic aromatic hydrocarbons (PAHs) in different tissues. These investigations will provide insights into how environmental factors and biological processes affect pollutant storage in aquatic organisms.

Additionally, incorporating vitellogenin analysis could be crucial, as this liver-produced protein, important for ovarian development, can be significantly altered by environmental contaminants. Examining vitellogenin levels across sexes and pollutant exposure will enhance the understanding of contaminants’ impact on reproductive health and improve environmental health assessments.

Finally, our results highlight that somatic indexes, like HSI and GSI, are useful indicators of metabolic state and reproductive health, reflecting environmental stress and pollution effects.

Overall, this multidisciplinary approach, including condition indexes, provides a deep understanding of the relationship between reproductive biology and the health of European hake, offering insights into sustainable fishery management and environmental conservation.

## Figures and Tables

**Table 1:** Total Hg concentrations (mg kg^−1^ ww) detected in the muscle of *M. merluccius* as a function of sex and season. # = number of valid values; median = median of the values; mean = mean of the values; sd = standard deviation; min = minimum value; max = maximum value.

Sex	Season	#	Median	Mean	sd	Min	Max
F	Spring	5	0.137	0.158	0.081	0.073	0.287
F	Summer	10	0.182	0.196	0.095	0.073	0.374
F	Autumn	10	0.103	0.103	0.033	0.045	0.152
F	Winter	13	0.096	0.091	0.02	0.059	0.122
M	Spring	10	0.155	0.155	0.032	0.111	0.217
M	Summer	7	0.125	0.131	0.074	0.071	0.289
M	Autumn	10	0.117	0.130	0.061	0.072	0.267
M	Winter	9	0.112	0.127	0.040	0.085	0.202

**Table 2:** Total PAH concentrations (ng g^−1^ ww) detected in the muscle of *M. merluccius* as a function of sex and season. # = number of valid values; median = median of the values; mean = mean of the values; sd = standard deviation; min = minimum value; max = maximum value.

Sex	Season	#	Median	Mean	sd	Min	Max
F	Spring	15	15.317	16.152	6.885	5.890	30.680
F	Summer	17	16.238	17.078	6.863	8.462	36.833
F	Autumn	9	4.990	5.998	2.287	3.236	8.959
F	Winter	34	99.680	89.221	41.464	23.725	185.788
M	Spring	33	10.211	16.931	22.421	3.713	117.229
M	Summer	14	5.593	5.835	1.443	3.881	8.206
M	Autumn	19	5.495	6.084	1.929	2.704	9.450
M	Winter	10	69.775	70.844	41.542	20.380	129.278

**Table 3:** GSI as a function of sex and season. # = number of valid values; median = median of the values; mean = mean of the values; sd = standard deviation; min = minimum value; max = maximum value.

Sex	Season	#	Median	Mean	sd	Min	Max
F	Spring	15	0.684	1.157	1.286	0.247	4.534
F	Summer	17	2.126	3.545	3.295	0.345	11.232
F	Autumn	19	0.685	1.510	2.355	0.053	8.536
F	Winter	18	0.421	0.393	0.117	0.136	0.562
M	Spring	32	0.809	0.792	0.394	0.196	2.009
M	Summer	14	0.728	0.850	0.419	0.353	1.615
M	Autumn	17	0.182	0.252	0.155	0.062	0.583
M	Winter	6	0.244	0.239	0.080	0.128	0.323

**Table 4:** HSI as a function of sex and season. # = number of valid values; median = median of the values; mean = mean of the values; sd = standard deviation; min = minimum value; max = maximum value.

Sex	Season	#	Median	Mean	sd	Min	Max
F	Spring	6	1.636	1.6500	0.389	1.036	2.126
F	Summer	16	2.690	3.059	1.111	1.720	5.422
F	Autumn	19	3.187	3.087	1.189	1.242	5.688
F	Winter	18	2.820	2.879	0.867	1.082	4.272
M	Spring	32	1.941	2.398	1.103	0.856	4.797
M	Summer	14	2.152	2.481	0.852	1.393	3.939
M	Autumn	18	1.693	2.038	0.959	1.132	4.724
M	Winter	6	1.663	1.653	0.296	1.250	2.072

**Table 5:** Le Cren’s Kn index as a function of sex and season. # = number of valid values; median = median of the values; mean = mean of the values; sd = standard deviation; min = minimum value; max = maximum value.

Sex	Season	#	Median	Mean	sd	Min	Max
F	Spring	15	1.098	1.084	0.085	0.921	1.233
F	Summer	17	1.196	1.159	0.079	1.011	1.262
F	Autumn	19	1.105	1.098	0.109	0.874	1.328
F	Winter	34	1.136	1.116	0.092	0.918	1.242
M	Spring	33	1.085	1.097	0.080	0.969	1.324
M	Summer	14	1.126	1.124	0.119	0.933	1.356
M	Autumn	17	1.050	1.065	0.091	0.912	1.284
M	Winter	11	1.037	1.097	0.149	0.985	1.485

**Table 6:** Kendall’s tau correlation matrix for the six numerical variables. The bold figures are statistically significant values.

	Lipids	Tot PAHs	GSI	HSI	Le Cren CF	THg
Lipids	1.000	−0.106	**0.254**	−0.072	−0.078	**0.447**
Tot PAHs		1.000	−0.079	0.071	−0.004	−0.175
GSI			1.000	**0.255**	0.154	**0.288**
HSI				1.000	**0.268**	−0.169
Le Cren CF					1.000	−0.046
THg						1.000

## Data Availability

Upon request due to restrictions, e.g., privacy or ethical.

## Supplementary Materials

The following supporting information can be downloaded at: https://www.mdpi.com/article/10.3390/ani14202938/s1, Table S1: Concentrations of Total Mercury (THg) in muscle tissues of Merluccius merluccius samples from the Adriatic Sea. Sex, biometric data of investigated fish. n, number of fish samples; mean value ± standard deviation; range (min–max) of length, weight, THg; Table S2: Concentrations of Total PAHs in muscle tissues of Merluccius merluccius samples from the Adriatic Sea. Sex, biometric data of investigated fish. n, number of fish samples; mean value ± standard deviation; range (min–max) of length, weight, PAHs; Table S3: Omnibus two-way ANOVA results and post hoc comparisons of Total Hg in relations to the Season and Sex factors along with their interaction; Table S4: Omnibus two-way ANOVA results and post hoc comparisons of Total PAHs in relations to the Season and Sex factors along with their interaction; Table S5: Omnibus two-way ANOVA results and post hoc comparisons of Lipids in relations to the Season and Sex factors along with their interaction; Table S6: Omnibus two-way ANOVA results and post hoc comparisons of GSI index in relations to the Season and Sex factors along with their interaction; Table S7: Omnibus two-way ANOVA results and post hoc comparisons of HSI index in relations to the Season and Sex factors along with their interaction; Table S8: Omnibus two-way ANOVA results and post hoc comparisons of Le Cren CF index in relations to the Season and Sex factors along with their interaction; Table S9: Spring/Female Kendall’s tau correlation matrix for the six numerical variables. Bold figures are statistically significant values; Table S10: Spring/Male Kendall’s tau correlation matrix for the six numerical variables. Bold figures are statistically significant values; Table S11: Summer/Female Kendall’s tau correlation matrix for the six numerical variables. Bold figures are statistically significant values; Table S12: Summer/Male Kendall’s tau correlation matrix for the six numerical variables. Bold figures are statistically significant values; Table S13: Autumn/Female Kendall’s tau correlation matrix for the six numerical variables. Bold figures are statistically significant values; Table S14: Autumn/Male Kendall’s tau correlation matrix for the six numerical variables. Bold figures are statistically significant values; Table S15: Winter/Female Kendall’s tau correlation matrix for the six numerical variables. Bold figures are statistically significant values; Table S16: Winter/Male Kendall’s tau correlation matrix for the six numerical variables. Bold figures are statistically significant values.
