# Supplementary material for: Influence of Contaminants Mercury and PAHs on Somatic Indexes of the European Hake (Merluccius merluccius, L. 1758)
Source: Animals (Basel). 2024 Oct 11;14(20):2938. doi: 10.3390/ani14202938 (PMC11503758; doi:10.3390/ani14202938)
Supplement: Supplementary file 1 [file animals-14-02938-s001.zip › animals-3153956-supplementary.pdf]

Supplemetary Materials

Table S1. Concentrations of Total Mercury (THg) in muscle tissues of *Merluccius merluccius* samples from the Adriatic Sea. Sex, biometric data of investigated fish. *n*, number of fish samples; mean value ± standard deviation; range (min–max) of length, weight, THg.

Sampling season	Sex	n	Weight, g Mean±sd	Length, cm Mean±sd	THg, mg kg $^{-1}$ w.w. Mean±sd	THg, mg kg $^{-1}$ w.w. (min-max)
Spring 2018	All	15	79±39	22±4	0.15±0.05	0.07-0.29
	F	5	73±40	21±4	0.16±0.08	0.07-0.29
	M	10	82±40	22±4	0.15±0.03	0.11-0.22
Summer 2018	All	17	166±68	27±4	0.17±0.09	0.07-0.37
	F	10	203±66	29±4	0.19±0.09	0.07-0.37
	M	7	135±14	25±1	0.13±0.07	0.07-0.29
Autumn 2018	All	20	97±66	21±5	0.12±0.05	0.04-0.27
	F	10	114±69	24±6	0.10±0.03	0.03-0.15
	M	10	79±61	21±5	0.13±0.06	0.07-0.27
Winter 2019	All	22	91±72	22±5	0.10±0.03	0.06-0.20
	F	13	126±76	24±5	0.09±0.02	0.06-0.12
	M	9	42±9	18±1	0.13±0.04	0.08-0.20

Sex	n	Weight, g Mean±sd	Length, cm Mean±sd	THg, mg kg $^{-1}$ w.w. Mean±sd	THg, mg kg $^{-1}$ w.w. (min-max)
All	74	107±71	23±5	0.13± 0.06	0.04-0.37
F	38	117±71	24±5	0.13±0.07	0.04-0.37
M	36	106±71	23±5	0.14±0.05	0.07-0.29

Table S2. Concentrations of Total PAHs in muscle tissues of *Merluccius merluccius* samples from the Adriatic Sea. Sex, biometric data of investigated fish. *n*, number of fish samples; mean value ± standard deviation; range (min–max) of length, weight, PAHs.

Sampling season	Sex	n	Weight, g Mean±sd	Length, cm Mean±sd	PAH, ng g $^{-1}$ w.w Mean±sd	PAH, ng g $^{-1}$ w.w (min-max)
Spring 2018	All	48	138±133	25±7	16.69±18.88	3.71-117.23
	F	15	260±179	30±9	16.15±6.88	5.89-30.68
	M	33	82±41	22±3	16.93±22.42	3.71-117.23
Summer 2018	All	31	166±70	27±4	12.00±7.64	3.88-36.83
	F	17	211±65	30±3	17.08±6.86	8.46-36.83
	M	14	110±18	24±18	5.83±1.44	3.88-8.21
Autumn 2018	All	28	110±99	23±7	6.06±2.01	2.70-9.45
	F	9	233±39	31±2	6.00±2.29	3.24-8.96
	M	19	48±46	18±4	6.08±1.93	2.70-9.45
Winter 2019	All	44	115±79	24±6	85.04±41.73	20.38-185.79
	F	34	137±77	25±5	89.22±41.46	23.72-185.79
	M	10	39±9	18±1	70.84±41.54	20.38-129.28

Sex	n	Weight, g Mean±sd	Length, cm Mean±sd	PAH, ng g $^{-1}$ w.w Mean±sd	PAH, ng g $^{-1}$ w.w (min-max)
All	151	132±102	24±6	33.1±40.60	2.70-185.79
F	75	139±103	25±6	48.27±46.98	3.24-185.79
M	76	125±102	24±6	19.27±29.28	2.70-129.28

Table S3. Omnibus two-way ANOVA results and post hoc comparisons of Total Hg in relations to the Season and Sex factors along with their interaction.

```
## Analysis of Variance of Aligned Rank Transformed Data
##
## Table Type: Anova Table (Type III tests)
## Model: No Repeated Measures (lm)
## Response: art(Hg)
##
##                Df Df.res  F value    Pr(>F)
## 1 Season        3     66 5.624749 0.0017031 **
## 2 SEX           1     66 0.095045 0.7588299
## 3 Season:SEX    3     66 3.123907 0.0316595  *
## ---
## Signif. codes:   0 '***' 0.001 '**' 0.01 '*' 0.05 '.' 0.1 ' ' 1
```

```
marginal <- art.con(m.art, "Season:SEX")
marginal
```

```
##  contrast            estimate    SE df t.ratio p.value
##  Autumn,F - Autumn,M    -7.60  8.38 66  -0.907  0.9844
##  Autumn,F - Spring,F   -16.60 10.26 66  -1.618  0.7379
##  Autumn,F - Spring,M   -23.60  8.38 66  -2.817  0.1082
##  Autumn,F - Summer,F   -25.50  8.38 66  -3.044  0.0624
##  Autumn,F - Summer,M    -5.69  9.23 66  -0.616  0.9985
##  Autumn,F - Winter,F     9.68  7.88 66   1.228  0.9205
##  Autumn,F - Winter,M   -10.51  8.61 66  -1.221  0.9227
##  Autumn,M - Spring,F    -9.00 10.26 66  -0.877  0.9871
##  Autumn,M - Spring,M   -16.00  8.38 66  -1.910  0.5492
##  Autumn,M - Summer,F   -17.90  8.38 66  -2.137  0.4030
##  Autumn,M - Summer,M     1.91  9.23 66   0.207  1.0000
##  Autumn,M - Winter,F    17.28  7.88 66   2.193  0.3694
##  Autumn,M - Winter,M    -2.91  8.61 66  -0.338  1.0000
##  Spring,F - Spring,M    -7.00 10.26 66  -0.682  0.9972
##  Spring,F - Summer,F    -8.90 10.26 66  -0.867  0.9880
##  Spring,F - Summer,M    10.91 10.97 66   0.995  0.9736
##  Spring,F - Winter,F    26.28  9.86 66   2.666  0.1518
##  Spring,F - Winter,M     6.09 10.45 66   0.583  0.9990
##  Spring,M - Summer,F    -1.90  8.38 66  -0.227  1.0000
##  Spring,M - Summer,M    17.91  9.23 66   1.941  0.5289
##  Spring,M - Winter,F    33.28  7.88 66   4.223  0.0018
##  Spring,M - Winter,M    13.09  8.61 66   1.521  0.7936
##  Summer,F - Summer,M    19.81  9.23 66   2.146  0.3971
##  Summer,F - Winter,F    35.18  7.88 66   4.465  0.0008
##  Summer,F - Winter,M    14.99  8.61 66   1.742  0.6606
##  Summer,M - Winter,F    15.36  8.78 66   1.749  0.6555
##  Summer,M - Winter,M    -4.83  9.44 66  -0.511  0.9996
##  Winter,F - Winter,M   -20.19  8.12 66  -2.485  0.2199
```

Table S4. Omnibus two-way ANOVA results and post hoc comparisons of Total PAHs in relations to the Season and Sex factors along with their interaction.

```
## Analysis of Variance of Aligned Rank Transformed Data
##
## Table Type: Anova Table (Type III tests)
## Model: No Repeated Measures (lm)
## Response: art(Tot_pah)
##
##                 Df Df.res F value                  Pr(>F)
## 1 Season         3    143  90.573 < 0.000000000000000222 ***
## 2 SEX            1    143  50.798      0.000000000046482 ***
## 3 Season:SEX     3    143   6.336             0.00045944 ***
## ---
## Signif. codes:   0 '***' 0.001 '**' 0.01 '*' 0.05 '.' 0.1 ' ' 1

marginal <- art.con(m.art, "Season:SEX")
marginal
```

```
##  contrast            estimate   SE  df t.ratio p.value
##  Autumn,F - Autumn,M   -0.661 8.80 143  -0.075  1.0000
##  Autumn,F - Spring,F  -50.622 9.17 143  -5.521  <.0001
##  Autumn,F - Spring,M  -34.556 8.18 143  -4.226  0.0011
##  Autumn,F - Summer,F  -54.438 8.96 143  -6.073  <.0001
##  Autumn,F - Summer,M    1.230 9.29 143   0.132  1.0000
##  Autumn,F - Winter,F -100.732 8.15 143 -12.358  <.0001
##  Autumn,F - Winter,M  -93.556 9.99 143  -9.364  <.0001
##  Autumn,M - Spring,F  -49.961 7.51 143  -6.652  <.0001
##  Autumn,M - Spring,M  -33.895 6.26 143  -5.413  <.0001
##  Autumn,M - Summer,F  -53.777 7.26 143  -7.408  <.0001
##  Autumn,M - Summer,M    1.891 7.66 143   0.247  1.0000
##  Autumn,M - Winter,F -100.071 6.23 143 -16.067  <.0001
##  Autumn,M - Winter,M  -92.895 8.50 143 -10.935  <.0001
##  Spring,F - Spring,M   16.067 6.77 143   2.373  0.2629
##  Spring,F - Summer,F   -3.816 7.70 143  -0.495  0.9997
##  Spring,F - Summer,M   51.852 8.08 143   6.417  <.0001
##  Spring,F - Winter,F  -50.110 6.74 143  -7.435  <.0001
##  Spring,F - Winter,M  -42.933 8.88 143  -4.836  0.0001
##  Spring,M - Summer,F  -19.882 6.49 143  -3.063  0.0519
##  Spring,M - Summer,M   35.786 6.94 143   5.160  <.0001
##  Spring,M - Winter,F  -66.176 5.31 143 -12.454  <.0001
##  Spring,M - Winter,M  -59.000 7.85 143  -7.517  <.0001
##  Summer,F - Summer,M   55.668 7.85 143   7.093  <.0001
##  Summer,F - Winter,F  -46.294 6.46 143  -7.167  <.0001
##  Summer,F - Winter,M  -39.118 8.67 143  -4.514  0.0003
##  Summer,M - Winter,F -101.962 6.91 143 -14.766  <.0001
##  Summer,M - Winter,M  -94.786 9.00 143 -10.528  <.0001
##  Winter,F - Winter,M    7.176 7.82 143   0.917  0.9840
```

Table S5. Omnibus two-way ANOVA results and post hoc comparisons of Lipids in relations to the Season and Sex factors along with their interaction.

```
## Analysis of Variance of Aligned Rank Transformed Data
##
## Table Type: Anova Table (Type III tests)
## Model: No Repeated Measures (lm)
## Response: art(Lipid)
##
##                Df Df.res F value                 Pr(>F)
## 1 Season        3    134  56.796 < 0.000000000000000222 ***
## 2 SEX           1    134 105.164 < 0.000000000000000222 ***
## 3 Season:SEX    3    134 113.647 < 0.000000000000000222 ***
## ---
## Signif. codes:   0 '***' 0.001 '**' 0.01 '*' 0.05 '.' 0.1 ' ' 1
```

```
marginal <- art.con(m.art, "Season:SEX")
marginal
```

```
##   contrast            estimate    SE  df t.ratio p.value
##   Autumn,F - Autumn,M    9.579  8.94 134   1.072  0.9615
##   Autumn,F - Spring,F  -19.500 11.64 134  -1.676  0.7029
##   Autumn,F - Spring,M  -32.788  8.30 134  -3.949  0.0031
##   Autumn,F - Summer,F  -31.824  9.10 134  -3.496  0.0144
##   Autumn,F - Summer,M   33.500  9.43 134   3.551  0.0121
##   Autumn,F - Winter,F   49.147  8.28 134   5.937  <.0001
##   Autumn,F - Winter,M  -36.900 10.15 134  -3.637  0.0091
##   Autumn,M - Spring,F  -29.079 10.34 134  -2.812  0.1007
##   Autumn,M - Spring,M  -42.367  6.36 134  -6.662  <.0001
##   Autumn,M - Summer,F  -41.402  7.37 134  -5.616  <.0001
##   Autumn,M - Summer,M   23.921  7.78 134   3.076  0.0505
##   Autumn,M - Winter,F   39.568  6.32 134   6.256  <.0001
##   Autumn,M - Winter,M  -46.479  8.63 134  -5.388  <.0001
##   Spring,F - Spring,M  -13.288  9.80 134  -1.356  0.8752
##   Spring,F - Summer,F  -12.324 10.49 134  -1.175  0.9376
##   Spring,F - Summer,M   53.000 10.77 134   4.919  0.0001
##   Spring,F - Winter,F   68.647  9.78 134   7.021  <.0001
##   Spring,F - Winter,M  -17.400 11.40 134  -1.526  0.7922
##   Spring,M - Summer,F    0.964  6.59 134   0.146  1.0000
##   Spring,M - Summer,M   66.288  7.04 134   9.412  <.0001
##   Spring,M - Winter,F   81.935  5.40 134  15.184  <.0001
##   Spring,M - Winter,M   -4.112  7.97 134  -0.516  0.9996
##   Summer,F - Summer,M   65.324  7.97 134   8.197  <.0001
##   Summer,F - Winter,F   80.971  6.56 134  12.345  <.0001
##   Summer,F - Winter,M   -5.076  8.80 134  -0.577  0.9991
##   Summer,M - Winter,F   15.647  7.01 134   2.231  0.3400
##   Summer,M - Winter,M  -70.400  9.14 134  -7.700  <.0001
##   Winter,F - Winter,M  -86.047  7.94 134 -10.832  <.0001
```

Table S6. Omnibus two-way ANOVA results and post hoc comparisons of GSI index in relations to the Season and Sex factors along with their interaction.

```
## Analysis of Variance of Aligned Rank Transformed Data
##
## Table Type: Anova Table (Type III tests)
## Model: No Repeated Measures (lm)
## Response: art(GSI)
##
##                Df Df.res F value        Pr(>F)
## 1 Season        3    130  10.599 0.00000278063 ***
## 2 SEX           1    130  31.193 0.00000013084 ***
## 3 Season:SEX    3    130  13.369 0.00000011673 ***
## ---
## Signif. codes:   0 '***' 0.001 '**' 0.01 '*' 0.05 '.' 0.1 ' ' 1
```

```
marginal <- art.con(m.art, "Season:SEX")
marginal
```

```
##  contrast             estimate    SE  df t.ratio p.value
##  Autumn,F - Autumn,M    53.533 10.51 130   5.095 <.0001
##  Autumn,F - Spring,F    -0.193 10.87 130  -0.018 1.0000
##  Autumn,F - Spring,M    -2.526  9.12 130  -0.277 1.0000
##  Autumn,F - Summer,F   -25.526 10.51 130  -2.429 0.2363
##  Autumn,F - Summer,M    -7.598 11.09 130  -0.685 0.9973
##  Autumn,F - Winter,F    33.529 10.35 130   3.239 0.0319
##  Autumn,F - Winter,M    58.140 14.74 130   3.945 0.0032
##  Autumn,M - Spring,F   -53.725 11.15 130  -4.819 0.0001
##  Autumn,M - Spring,M   -56.059  9.45 130  -5.935 <.0001
##  Autumn,M - Summer,F   -79.059 10.80 130  -7.323 <.0001
##  Autumn,M - Summer,M   -61.130 11.36 130  -5.382 <.0001
##  Autumn,M - Winter,F   -20.003 10.64 130  -1.879 0.5676
##  Autumn,M - Winter,M     4.608 14.95 130   0.308 1.0000
##  Spring,F - Spring,M    -2.333  9.85 130  -0.237 1.0000
##  Spring,F - Summer,F   -25.333 11.15 130  -2.272 0.3170
##  Spring,F - Summer,M    -7.405 11.70 130  -0.633 0.9983
##  Spring,F - Winter,F    33.722 11.00 130   3.065 0.0523
##  Spring,F - Winter,M    58.333 15.20 130   3.837 0.0047
##  Spring,M - Summer,F   -23.000  9.45 130  -2.435 0.2337
##  Spring,M - Summer,M    -5.071 10.09 130  -0.503 0.9996
##  Spring,M - Winter,F    36.056  9.27 130   3.888 0.0039
##  Spring,M - Winter,M    60.667 14.00 130   4.333 0.0008
##  Summer,F - Summer,M    17.929 11.36 130   1.578 0.7623
##  Summer,F - Winter,F    59.056 10.64 130   5.548 <.0001
##  Summer,F - Winter,M    83.667 14.95 130   5.598 <.0001
##  Summer,M - Winter,F    41.127 11.22 130   3.667 0.0083
##  Summer,M - Winter,M    65.738 15.36 130   4.280 0.0009
##  Winter,F - Winter,M    24.611 14.84 130   1.659 0.7135
```

Table S7. Omnibus two-way ANOVA results and post hoc comparisons of HSI index in relations to the Season and Sex factors along with their interaction.

```
## Analysis of Variance of Aligned Rank Transformed Data
##
## Table Type: Anova Table (Type III tests)
## Model: No Repeated Measures (lm)
## Response: art(HSI)
##
##                 Df Df.res F value    Pr(>F)
## 1 Season        3    121  1.0413 0.3769110
## 2 SEX           1    121 10.7757 0.0013445 **
## 3 Season:SEX    3    121  5.5052 0.0014056 **
## ---
## Signif. codes:   0 '***' 0.001 '**' 0.01 '*' 0.05 '.' 0.1 ' ' 1
```

```
marginal <- art.con(m.art, "Season:SEX")
marginal
```

```
##   contrast            estimate    SE  df t.ratio p.value
##   Autumn,F - Autumn,M   39.143 11.19 121   3.497  0.0147
##   Autumn,F - Spring,F   51.088 15.94 121   3.206  0.0355
##   Autumn,F - Spring,M   24.296  9.86 121   2.465  0.2206
##   Autumn,F - Summer,F   -0.266 11.55 121  -0.023  1.0000
##   Autumn,F - Summer,M   17.207 11.99 121   1.436  0.8389
##   Autumn,F - Winter,F    2.199 11.19 121   0.196  1.0000
##   Autumn,F - Winter,M   51.921 15.94 121   3.258  0.0305
##   Autumn,M - Spring,F   11.944 16.04 121   0.745  0.9954
##   Autumn,M - Spring,M  -14.847 10.03 121  -1.481  0.8161
##   Autumn,M - Summer,F  -39.410 11.69 121  -3.371  0.0218
##   Autumn,M - Summer,M  -21.937 12.13 121  -1.809  0.6153
##   Autumn,M - Winter,F  -36.944 11.34 121  -3.257  0.0306
##   Autumn,M - Winter,M   12.778 16.04 121   0.797  0.9930
##   Spring,F - Spring,M  -26.792 15.14 121  -1.770  0.6415
##   Spring,F - Summer,F  -51.354 16.29 121  -3.152  0.0414
##   Spring,F - Summer,M  -33.881 16.60 121  -2.040  0.4595
##   Spring,F - Winter,F  -48.889 16.04 121  -3.048  0.0553
##   Spring,F - Winter,M    0.833 19.65 121   0.042  1.0000
##   Spring,M - Summer,F  -24.562 10.42 121  -2.357  0.2721
##   Spring,M - Summer,M   -7.089 10.90 121  -0.650  0.9980
##   Spring,M - Winter,F  -22.097 10.03 121  -2.204  0.3568
##   Spring,M - Winter,M   27.625 15.14 121   1.825  0.6046
##   Summer,F - Summer,M   17.473 12.45 121   1.403  0.8542
##   Summer,F - Winter,F    2.465 11.69 121   0.211  1.0000
##   Summer,F - Winter,M   52.188 16.29 121   3.204  0.0358
##   Summer,M - Winter,F  -15.008 12.13 121  -1.238  0.9190
##   Summer,M - Winter,M   34.714 16.60 121   2.091  0.4269
##   Winter,F - Winter,M   49.722 16.04 121   3.100  0.0480
```

Table S8. Omnibus two-way ANOVA results and post hoc comparisons of Le Cren CF index in relations to the Season and Sex factors along with their interaction.

```
## Analysis of Variance of Aligned Rank Transformed Data
##
## Table Type: Anova Table (Type III tests)
## Model: No Repeated Measures (lm)
## Response: art(Kn_Le_Cren)
##
##                Df Df.res F value   Pr(>F)
## 1 Season        3    152  2.5282 0.059498 .
## 2 SEX           1    152  5.7602 0.017603 *
## 3 Season:SEX    3    152  1.1211 0.342451
## ---
## Signif. codes:   0 '***' 0.001 '**' 0.01 '*' 0.05 '.' 0.1 ' ' 1
```

```
marginal <- art.con(m.art, "Season:SEX")
marginal
```

```
##   contrast            estimate   SE  df t.ratio p.value
##   Autumn,F - Autumn,M    22.22 15.1 152   1.473  0.8203
##   Autumn,F - Spring,F     7.56 15.6 152   0.485  0.9997
##   Autumn,F - Spring,M     5.96 13.0 152   0.458  0.9998
##   Autumn,F - Summer,F   -27.52 15.1 152  -1.825  0.6042
##   Autumn,F - Summer,M    -7.11 15.9 152  -0.447  0.9998
##   Autumn,F - Winter,F    -8.46 12.9 152  -0.654  0.9980
##   Autumn,F - Winter,M    20.94 17.1 152   1.224  0.9237
##   Autumn,M - Spring,F   -14.66 16.0 152  -0.916  0.9841
##   Autumn,M - Spring,M   -16.26 13.5 152  -1.206  0.9291
##   Autumn,M - Summer,F   -49.74 15.5 152  -3.210  0.0338
##   Autumn,M - Summer,M   -29.32 16.3 152  -1.799  0.6218
##   Autumn,M - Winter,F   -30.68 13.4 152  -2.286  0.3079
##   Autumn,M - Winter,M    -1.28 17.5 152  -0.073  1.0000
##   Spring,F - Spring,M    -1.61 14.1 152  -0.114  1.0000
##   Spring,F - Summer,F   -35.08 16.0 152  -2.192  0.3622
##   Spring,F - Summer,M   -14.67 16.8 152  -0.874  0.9880
##   Spring,F - Winter,F   -16.02 14.0 152  -1.144  0.9458
##   Spring,F - Winter,M    13.38 17.9 152   0.746  0.9954
##   Spring,M - Summer,F   -33.47 13.5 152  -2.482  0.2110
##   Spring,M - Summer,M   -13.06 14.4 152  -0.907  0.9851
##   Spring,M - Winter,F   -14.41 11.0 152  -1.306  0.8954
##   Spring,M - Winter,M    14.98 15.7 152   0.953  0.9801
##   Summer,F - Summer,M    20.41 16.3 152   1.252  0.9146
##   Summer,F - Winter,F    19.06 13.4 152   1.421  0.8465
##   Summer,F - Winter,M    48.46 17.5 152   2.773  0.1098
##   Summer,M - Winter,F    -1.35 14.3 152  -0.094  1.0000
##   Summer,M - Winter,M    28.05 18.2 152   1.541  0.7839
##   Winter,F - Winter,M    29.40 15.7 152   1.876  0.5692
```

Table S9. Spring/Female Kendall's tau correlation matrix for the six numerical variables. Bold figures are statistically significant values.

	Lipids	Tot PAHs	GSI	HSI	Le Cren CF	THg
Lipids	-	-	-	-	-	-
Tot PAHs		1.000	0.429	-0.200	-0.162	-0.600
GSI			1.000	-0.467	-0.124	0.200
HSI				1.000	0.733	-0.200
Le Cren CF					1.000	-0.200
THg						1.000

Table S10. Spring/Male Kendall's tau correlation matrix for the six numerical variables. Bold figures are statistically significant values.

	Lipids	Tot PAHs	GSI	HSI	Le Cren CF	THg
Lipids	-	-0.390	0.522	0.398	0.066	-
Tot PAHs		1.000	0.048	-0.0040	-0.049	-0.422
GSI			1.000	0.402	0.226	0.067
HSI				1.000	0.190	-0.111
Le Cren CF					1.000	-0.156
THg						1.000

Table S11. Summer/Female Kendall's tau correlation matrix for the six numerical variables. Bold figures are statistically significant values.

	Lipids	Tot PAHs	GSI	HSI	Le Cren CF	THg
Lipids	-	0.148	0.698	-0.047	-0.106	0.423
Tot PAHs		1.000	-0.029	-0.083	-0.184	-0.022
GSI			1.000	0.017	-0.066	0.333
HSI				1.000	0.477	-0.278
Le Cren CF					1.000	-0.200
THg						1.000

Table S12. Summer/Male Kendall's tau correlation matrix for the six numerical variables. Bold figures are statistically significant values.

	Lipids	Tot PAHs	GSI	HSI	Le Cren CF	THg
Lipids	-	-	-	-	-	-
Tot PAHs		1.000	0.050	-0.099	-0.231	-0.048
GSI			1.000	-0.648	0.055	0.238
HSI				1.000	0.121	-0.429
Le Cren CF					1.000	0.048
THg						1.000

Table S13. Autumn/Female Kendall's tau correlation matrix for the six numerical variables. Bold figures are statistically significant values.

	Lipids	Tot PAHs	GSI	HSI	Le Cren CF	THg
Lipids	-	-	-	-	-	-
Tot PAHs		-	-	-	-	-
GSI			1.000	0.251	0.287	-
HSI				1.000	0.474	-
Le Cren CF					1.000	-
THg						-

Table S14. Autumn/Male Kendall's tau correlation matrix for the six numerical variables. Bold figures are statistically significant values.

	Lipids	Tot PAHs	GSI	HSI	Le Cren CF	THg
Lipids	1.000	0.509	0.395	0.476	-0.044	0.314
Tot PAHs		1.000	0.200	0.500	0.050	0.611
GSI			1.000	0.317	-0.103	-0.067
HSI				1.000	0.237	-0.244
Le Cren CF					1.000	-0.067
THg						-

Table S15. Winter/Female Kendall's tau correlation matrix for the six numerical variables. Bold figures are statistically significant values.

	Lipids	Tot PAHs	GSI	HSI	Le Cren CF	THg
Lipids	1.000	-0.106	-	-	-0.243	-
Tot PAHs		1.000	0.066	-0.079	-0.150	-0.051
GSI			1.000	0.329	-0.303	-0.214
HSI				1.000	0.053	-0.286
Le Cren CF					1.000	-0.103
THg						-

Table S16. Winter/Male Kendall's tau correlation matrix for the six numerical variables. Bold figures are statistically significant values.

	Lipids	Tot PAHs	GSI	HSI	Le Cren CF	THg
Lipids	1.000	-0.183	-	-	-0.243	0.189
Tot PAHs		1.000	0.067	-0.333	-0.156	0.214
GSI			1.000	0.600	-0.067	0.000
HSI				1.000	-0.467	0.000
Le Cren CF					1.000	0.111
THg						-
